# Anticosti Island: a hot spot for *Neospondylis upiformis* (Coleoptera: Cerambycidae) in eastern Canada?

**DOI:** 10.3897/BDJ.6.e25553

**Published:** 2018-07-19

**Authors:** Christian Hébert, Serge Laplante, Mario Fréchette, Luc Jobin

**Affiliations:** 1 Natural Resources Canada, Canadian Forest Service, Laurentian Forestry Centre, 1055 du P.E.P.S., P.O. Box 10380, Stn. Sainte-Foy, G1V 4C7, Québec City, Canada; 2 Canadian National Collection of Insects, Arachnids and Nematodes, Agriculture and Agri-Food Canada, Central Experimental Farm, KW Neatby Bldg., 960 Carling Ave., K1A 0C6, Ottawa, Canada; 3 Ministère de l'Agriculture, des Pêcheries et de l'Alimentation du Québec, Collection d'insectes du Québec, Complexe scientifique, 2700 rue Einstein, bur. D2.370A, G1P 3W8, Québec City, Canada

**Keywords:** Anticosti island, insect collections, inventory data, Lindgren funnel traps, hot spot

## Abstract

**Background:**

During an inventory of insect diversity on Anticosti Island in 1993, we caught unprecedented numbers of *Neospondylis upiformis* (Mannerheim), a longhorned beetle rarely observed in eastern North America. All specimens were caught using 12-funnel Lindgren traps baited with 95% ethanol and α-pinene. This longhorned beetle was captured again in 2007 on Anticosti with the same traps. Other than that, seven specimens of *N. upiformis* were caught elsewhere in Quebec between 1993 and 2015. Only 14 specimens were found in the 45 most important insect collections of the province, the most recent specimen dating back to 1964.

**New information:**

At least 90% of the captures came from old-growth balsam fir stands of the south-central part of the island. Seasonal flight activity ranged from early June to late July, but adult captures peaked in early July. Results suggest that Anticosti Island might be a hot spot for *N. upiformis* in eastern North America, particularly in its south-central part where old-growth balsam fir forests still exist.

## Introduction

During a general inventory of beetle diversity carried out in 1993 on Anticosti Island, Quebec, Canada, we caught unprecedented numbers of *Neospondylis upiformis* (Mannerheim) (Coleoptera: Cerambycidae, Spondylidinae), a species formerly included in the genus *Spondylis* Fabricius (Sama 2005), in baited Lindgren multiple-funnel traps. Very few *N. upiformis* were captured with the same trap in other projects thereafter, except in 2007, again on Anticosti Island, where we conducted a second survey in order to validate our 1993 results.

Spondylidinae is a subfamily of Cerambycidae in which adults have short antennae (Chemsak 1996; Yanega 1996; Bousquet et al. 2017). *Neospondylis upiformis* is known as a common species in western North America, but it is rare in the east (Chemsak 1996) where its distribution was known to reach only the province of Quebec (McNamara 1991; Laplante et al. 1991) until the mid-2000, when four adults were collected in Newfoundland (Smith and Hurley 2005), 17 in New Brunswick (Webster et al. 2012; Webster pers. comm. 2018; Sweeney pers. comm. 2018) and 12 in Nova Scotia (Majka and Ogden 2010). The life history of *N. upiformis* is poorly known. According to Chemsak (1996), adults are diurnal and fly between May and September, while Gosling (1973) reported adult captures between 12 June and 25 July in the Great Lakes region. The only report on immature stages was published by Gardiner (1970) who found several larvae and two pupae in two white spruce, *Picea glauca* (Moench) Voss, stumps that had been cut 2 years earlier in Alberta, Canada. Based on this study, Gardiner (1970) suggested a 2-year life cycle for *N. upiformis*. Gardiner (1970) also reported that larval galleries were observed in roots, sometimes more than 1 m from the stump and as much as 50 cm below the soil surface; larval galleries progressed along the roots towards the stump where pupation took place. According to Gardiner (1970), adults would be adapted for digging with their unusual large mandibles and terminal lamellae on the fore tibiae (Gardiner 1970). Moreover, we recently observed that adult mandibles are scoop-shaped and larger in females, which may facilitate digging.

In this paper, we compiled data on *N. upiformis* from various inventories carried out as a part of the biodiversity research programme of the Laurentian Forestry Centre of the Canadian Forest Service over the last 25 years and also from labelled specimens found in 45 of the most important insect collections in Quebec. We provide data on adult seasonal flight activity and update the distribution map of *N. upiformis*, suggesting that Anticosti Island might be a hot spot for this species in eastern North America.

## Materials and methods

**Figure 1.**
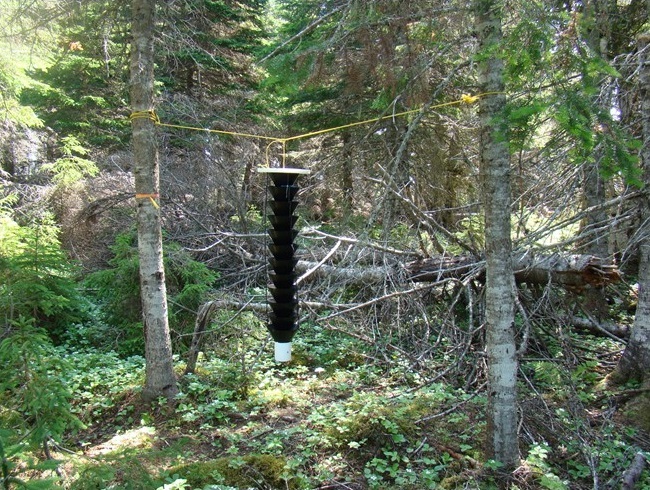
A 12-funnel Lindgren trap installed in 2007 at Lac McRae on Anticosti Island, Quebec, Canada.

Data were obtained from two sources: first, from field sampling and second, by compiling data from insect collections and from the recent literature. First, 12-funnel Lindgren traps (Lindgren 1983), baited with Ultra High Release ethanol (95%) and α-pinene produced by Phero Tech Inc. (Delta, British Columbia), were used in each of 45 stands (mainly coniferous) sampled across 10 projects carried out in various regions of the province of Quebec between 1993 and 2015. Site locations, general descriptions and sampling parameters are summarised in Table 1. Each trap was suspended at 2 m high on a rope placed between two healthy trees located at least 4 m apart to make sure that the trap was at least 2 m away from any other tree (Fig. 1). The collecting bottle was filled with 100 ml of 70% ethanol to kill and preserve insects. Samples were sorted in the laboratory and specimens were mounted, identified, sexed and counted. Vouchers were deposited at the Insectarium René-Martineau of the Laurentian Forestry Centre-Canadian Forest Service (LFC-CFS) and at the Collection d’insectes du Québec of the Ministère de Forêts, de la Faune et des Parcs du Québec. Data are stored at LFC-CFS in a data management system (MicroSIGEB) (Francoeur 2000).

Available temperature data were incomplete on Anticosti Island except in 2007, our last year of sampling on the island. Thus, we used the 2007 daily temperature averages from Havre Saint-Pierre and Cap-des-Rosiers, respectively on the north and south shores of the St. Lawrence River, to determine whether these data could be used as surrogates to express day-degree accumulation on Anticosti Island. In May 2007, degree-day accumulation on Anticosti Island followed very closely that of Havre Saint-Pierre but an average of Havre Saint-Pierre and Cap-des-Rosiers was a better fit in June and July (Suppl. material 1). We thus used daily averages of Havre Saint-Pierre in May and of both sites in June and July as surrogates for Anticosti Island in 1993 and 1998.

Finally, 45 insect collections, including the most important ones in Quebec, were visited by one of the authors (SL) to authenticate *N. upiformis* specimens and compile data on labels (location, collection date and collector).

## Taxon treatments

### Neospondylis upiformis

(Mannerheim, 1843) Sama, 2005

https://www.gbif.org/species/1143134

Spondylis upiformis Mannerheim, 1843

#### Materials

- **Type status:** Other material. **Occurrence:** recordNumber: 1993-3-1921; recordedBy: HEBECH01; individualCount: 1; sex: M; lifeStage: CI; preparations: pinned; disposition: in collection; **Taxon:** scientificNameID: CERASPONUPIF; family: Cerambycidae; taxonRank: Organism; scientificNameAuthorship: Neospondylis upiformis (Mannerheim 1843); **Location:** locationID: 9904; locality: Lac Metis; verbatimLatitude: 4818; verbatimLongitude: 6748; **Identification:** identifiedBy: FRECMA01; dateIdentified: 1997; **Event:** samplingProtocol: 12 funnel Lindgren traps, baited with 95% Ethanol and α-pinene; habitat: Mature balsam fir forest; **Record Level:** collectionID: CCFL; datasetName: Plan vert 1993
- **Type status:** Other material. **Occurrence:** occurrenceRemarks: Barcode of life, Sample ID LFCa-08-114; recordNumber: 1993-3-2073; recordedBy: HEBECH01; individualCount: 1; sex: F; lifeStage: CI; preparations: pinned; disposition: in collection; **Taxon:** scientificNameID: CERASPONUPIF; family: Cerambycidae; taxonRank: Organism; scientificNameAuthorship: Neospondylis upiformis (Mannerheim 1843); **Location:** locationID: 9904; locality: Lac Metis; verbatimLatitude: 4818; verbatimLongitude: 6748; **Identification:** identifiedBy: FRECMA01; dateIdentified: 1997; **Event:** samplingProtocol: 12 funnel Lindgren traps, baited with 95% Ethanol and α-pinene; habitat: Mature balsam fir forest; **Record Level:** collectionID: CCFL; datasetName: Plan vert 1993
- **Type status:** Other material. **Occurrence:** recordNumber: 1993-3-4269; recordedBy: HEBECH01; individualCount: 1; sex: M; lifeStage: CI; preparations: pinned; disposition: in collection; **Taxon:** scientificNameID: CERASPONUPIF; family: Cerambycidae; taxonRank: Organism; scientificNameAuthorship: Neospondylis upiformis (Mannerheim 1843); **Location:** locationID: 1530; locality: La loutre River; verbatimLatitude: 4947; verbatimLongitude: 6341; **Identification:** identifiedBy: FRECMA01; dateIdentified: 1997; **Event:** samplingProtocol: 12 funnel Lindgren traps, baited with 95% Ethanol and α-pinene; habitat: Old balsam fir fores; **Record Level:** collectionID: CCFL; datasetName: Anticosti 1993
- **Type status:** Other material. **Occurrence:** recordNumber: 1993-3-4284; recordedBy: HEBECH01; individualCount: 2; sex: F; lifeStage: CI; preparations: pinned; disposition: in collection; **Taxon:** scientificNameID: CERASPONUPIF; family: Cerambycidae; taxonRank: Organisme; scientificNameAuthorship: Neospondylis upiformis (Mannerheim 1843); **Location:** locationID: 1530; locality: La loutre River; verbatimLatitude: 4947; verbatimLongitude: 6341; **Identification:** identifiedBy: FRECMA01; dateIdentified: 1997; **Event:** samplingProtocol: 12 funnel Lindgren traps, baited with 95% Ethanol and α-pinene; habitat: Old balsam fir fores; **Record Level:** collectionID: CCFL; datasetName: Anticosti 1993
- **Type status:** Other material. **Occurrence:** recordNumber: 1993-3-4314; recordedBy: HEBECH01; individualCount: 2; sex: M; lifeStage: CI; preparations: pinned; disposition: in collection; **Taxon:** scientificNameID: CERASPONUPIF; family: Cerambycidae; taxonRank: Organism; scientificNameAuthorship: Neospondylis upiformis (Mannerheim 1843); **Location:** locationID: 1530; locality: Jupiter River road; verbatimLatitude: 4942; verbatimLongitude: 6327; **Identification:** identifiedBy: FRECMA01; dateIdentified: 1997; **Event:** samplingProtocol: 12 funnel Lindgren traps, baited with 95% Ethanol and α-pinene; habitat: Trembling aspen forest; **Record Level:** collectionID: CCFL; datasetName: Anticosti 1993
- **Type status:** Other material. **Occurrence:** recordNumber: 1993-3-4314; recordedBy: HEBECH01; individualCount: 1; sex: F; lifeStage: CI; preparations: pinned; disposition: in collection; **Taxon:** scientificNameID: CERASPONUPIF; family: Cerambycidae; taxonRank: Organism; scientificNameAuthorship: Neospondylis upiformis (Mannerheim 1843); **Location:** locationID: 1530; locality: Jupiter River road; verbatimLatitude: 4942; verbatimLongitude: 6327; **Identification:** identifiedBy: FRECMA01; dateIdentified: 1997; **Event:** samplingProtocol: 12 funnel Lindgren traps, baited with 95% Ethanol and α-pinene; habitat: Trembling aspen forest; **Record Level:** collectionID: CCFL; datasetName: Anticosti 1993
- **Type status:** Other material. **Occurrence:** recordNumber: 1993-3-4319; recordedBy: HEBECH01; individualCount: 1; sex: M; lifeStage: CI; preparations: pinned; disposition: in collection; **Taxon:** scientificNameID: CERASPONUPIF; family: Cerambycidae; taxonRank: Organism; scientificNameAuthorship: Neospondylis upiformis (Mannerheim 1843); **Location:** locationID: 1530; locality: Jupiter River road; verbatimLatitude: 4942; verbatimLongitude: 6327; **Identification:** identifiedBy: FRECMA01; dateIdentified: 1997; **Event:** samplingProtocol: 12 funnel Lindgren traps, baited with 95% Ethanol and α-pinene; habitat: Trembling aspen forest; **Record Level:** collectionID: CCFL; datasetName: Anticosti 1993
- **Type status:** Other material. **Occurrence:** recordNumber: 1993-3-4319; recordedBy: HEBECH01; individualCount: 3; sex: F; lifeStage: CI; preparations: pinned; disposition: in collection; **Taxon:** scientificNameID: CERASPONUPIF; family: Cerambycidae; taxonRank: Organism; scientificNameAuthorship: Neospondylis upiformis (Mannerheim 1843); **Location:** locationID: 1530; locality: Jupiter River road; verbatimLatitude: 4942; verbatimLongitude: 6327; **Identification:** identifiedBy: FRECMA01; dateIdentified: 1997; **Event:** samplingProtocol: 12 funnel Lindgren traps, baited with 95% Ethanol and α-pinene; habitat: Trembling aspen forest; **Record Level:** collectionID: CCFL; datasetName: Anticosti 1993
- **Type status:** Other material. **Occurrence:** recordNumber: 1993-3-4324; recordedBy: HEBECH01; individualCount: 1; sex: F; lifeStage: CI; preparations: pinned; disposition: in collection; **Taxon:** scientificNameID: CERASPONUPIF; family: Cerambycidae; taxonRank: Organism; scientificNameAuthorship: Neospondylis upiformis (Mannerheim 1843); **Location:** locationID: 1530; locality: Jupiter River road; verbatimLatitude: 4942; verbatimLongitude: 6327; **Identification:** identifiedBy: FRECMA01; dateIdentified: 1997; **Event:** samplingProtocol: 12 funnel Lindgren traps, baited with 95% Ethanol and α-pinene; habitat: Trembling aspen forest; **Record Level:** collectionID: CCFL; datasetName: Anticosti 1993
- **Type status:** Other material. **Occurrence:** recordNumber: 1993-3-4329; recordedBy: HEBECH01; individualCount: 1; sex: M; lifeStage: CI; preparations: pinned; disposition: in collection; **Taxon:** scientificNameID: CERASPONUPIF; family: Cerambycidae; taxonRank: Organism; scientificNameAuthorship: Neospondylis upiformis (Mannerheim 1843); **Location:** locationID: 1530; locality: Jupiter River road; verbatimLatitude: 4942; verbatimLongitude: 6327; **Identification:** identifiedBy: FRECMA01; dateIdentified: 1997; **Event:** samplingProtocol: 12 funnel Lindgren traps, baited with 95% Ethanol and α-pinene; habitat: Trembling aspen forest; **Record Level:** collectionID: CCFL; datasetName: Anticosti 1993
- **Type status:** Other material. **Occurrence:** recordNumber: 1993-3-4379; recordedBy: HEBECH01; individualCount: 1; sex: M; lifeStage: CI; preparations: pinned; disposition: in collection; **Taxon:** scientificNameID: CERASPONUPIF; family: Cerambycidae; taxonRank: Organism; scientificNameAuthorship: Neospondylis upiformis (Mannerheim 1843); **Location:** locationID: 1530; locality: Jupiter River road; verbatimLatitude: 4941; verbatimLongitude: 6327; **Identification:** identifiedBy: FRECMA01; dateIdentified: 1997; **Event:** samplingProtocol: 12 funnel Lindgren traps, baited with 95% Ethanol and α-pinene; habitat: Young black spruce forest; **Record Level:** collectionID: CCFL; datasetName: Anticosti 1993
- **Type status:** Other material. **Occurrence:** recordNumber: 1993-3-4384; recordedBy: HEBECH01; individualCount: 1; sex: M; lifeStage: CI; preparations: pinned; disposition: in collection; **Taxon:** scientificNameID: CERASPONUPIF; family: Cerambycidae; taxonRank: Organism; scientificNameAuthorship: Neospondylis upiformis (Mannerheim 1843); **Location:** locationID: 1530; locality: Jupiter River road; verbatimLatitude: 4941; verbatimLongitude: 6327; **Identification:** identifiedBy: FRECMA01; dateIdentified: 1997; **Event:** samplingProtocol: 12 funnel Lindgren traps, baited with 95% Ethanol and α-pinene; habitat: Young black spruce forest; **Record Level:** collectionID: CCFL; datasetName: Anticosti 1993
- **Type status:** Other material. **Occurrence:** recordNumber: 1993-3-4414; recordedBy: HEBECH01; individualCount: 2; sex: M; lifeStage: CI; preparations: pinned; disposition: in collection; **Taxon:** scientificNameID: CERASPONUPIF; family: Cerambycidae; taxonRank: Organism; scientificNameAuthorship: Neospondylis upiformis (Mannerheim 1843); **Location:** locationID: 1530; locality: Jupiter River road; verbatimLatitude: 4940; verbatimLongitude: 6327; **Identification:** identifiedBy: FRECMA01; dateIdentified: 1997; **Event:** samplingProtocol: 12 funnel Lindgren traps, baited with 95% Ethanol and α-pinene; habitat: Old-growth black spruce forest; **Record Level:** collectionID: CCFL; datasetName: Anticosti 1993
- **Type status:** Other material. **Occurrence:** recordNumber: 1993-3-4414; recordedBy: HEBECH01; individualCount: 1; sex: F; lifeStage: CI; preparations: pinned; disposition: in collection; **Taxon:** scientificNameID: CERASPONUPIF; family: Cerambycidae; taxonRank: Organism; scientificNameAuthorship: Neospondylis upiformis (Mannerheim 1843); **Location:** locationID: 1530; locality: Jupiter River road; verbatimLatitude: 4940; verbatimLongitude: 6327; **Identification:** identifiedBy: FRECMA01; dateIdentified: 1997; **Event:** samplingProtocol: 12 funnel Lindgren traps, baited with 95% Ethanol and α-pinene; habitat: Old-growth black spruce forest; **Record Level:** collectionID: CCFL; datasetName: Anticosti 1993
- **Type status:** Other material. **Occurrence:** recordNumber: 1993-3-4419; recordedBy: HEBECH01; individualCount: 1; sex: M; lifeStage: CI; preparations: pinned; disposition: in collection; **Taxon:** scientificNameID: CERASPONUPIF; family: Cerambycidae; taxonRank: Organism; scientificNameAuthorship: Neospondylis upiformis (Mannerheim 1843); **Location:** locationID: 1530; locality: Jupiter River road; verbatimLatitude: 4940; verbatimLongitude: 6327; **Identification:** identifiedBy: FRECMA01; dateIdentified: 1997; **Event:** samplingProtocol: 12 funnel Lindgren traps, baited with 95% Ethanol and α-pinene; habitat: Old-growth black spruce forest; **Record Level:** collectionID: CCFL; datasetName: Anticosti 1993
- **Type status:** Other material. **Occurrence:** recordNumber: 1993-3-4464; recordedBy: HEBECH01; individualCount: 10; sex: M; lifeStage: CI; preparations: pinned; disposition: in collection; **Taxon:** scientificNameID: CERASPONUPIF; family: Cerambycidae; taxonRank: Organism; scientificNameAuthorship: Neospondylis upiformis (Mannerheim 1843); **Location:** locationID: 1530; locality: Jupiter River; verbatimLatitude: 4931; verbatimLongitude: 6321; **Identification:** identifiedBy: FRECMA01; dateIdentified: 1997; **Event:** samplingProtocol: 12 funnel Lindgren traps, baited with 95% Ethanol and α-pinene; habitat: Old-growth balsam fir forest; **Record Level:** collectionID: CCFL; datasetName: Anticosti 1993
- **Type status:** Other material. **Occurrence:** recordNumber: 1993-3-4464; recordedBy: HEBECH01; individualCount: 6; sex: F; lifeStage: CI; preparations: pinned; disposition: in collection; **Taxon:** scientificNameID: CERASPONUPIF; family: Cerambycidae; taxonRank: Organism; scientificNameAuthorship: Neospondylis upiformis (Mannerheim 1843); **Location:** locationID: 1530; locality: Jupiter River; verbatimLatitude: 4931; verbatimLongitude: 6321; **Identification:** identifiedBy: FRECMA01; dateIdentified: 1997; **Event:** samplingProtocol: 12 funnel Lindgren traps, baited with 95% Ethanol and α-pinene; habitat: Old-growth balsam fir forest; **Record Level:** collectionID: CCFL; datasetName: Anticosti 1993
- **Type status:** Other material. **Occurrence:** recordNumber: 1993-3-4464; recordedBy: HEBECH01; individualCount: 5; sex: I; lifeStage: CI; disposition: missing; **Taxon:** scientificNameID: CERASPONUPIF; family: Cerambycidae; taxonRank: Organism; scientificNameAuthorship: Neospondylis upiformis (Mannerheim 1843); **Location:** locationID: 1530; locality: Jupiter River; verbatimLatitude: 4931; verbatimLongitude: 6321; **Identification:** identifiedBy: FRECMA01; dateIdentified: 1997; **Event:** samplingProtocol: 12 funnel Lindgren traps, baited with 95% Ethanol and α-pinene; habitat: Old-growth balsam fir forest; **Record Level:** datasetName: Anticosti 1993
- **Type status:** Other material. **Occurrence:** recordNumber: 1993-3-4469; recordedBy: HEBECH01; individualCount: 4; sex: M; lifeStage: CI; preparations: pinned; disposition: in collection; **Taxon:** scientificNameID: CERASPONUPIF; family: Cerambycidae; taxonRank: Organism; scientificNameAuthorship: Neospondylis upiformis (Mannerheim 1843); **Location:** locationID: 1530; locality: Jupiter River; verbatimLatitude: 4931; verbatimLongitude: 6321; **Identification:** identifiedBy: FRECMA01; dateIdentified: 1997; **Event:** samplingProtocol: 12 funnel Lindgren traps, baited with 95% Ethanol and α-pinene; habitat: Old-growth balsam fir forest; **Record Level:** collectionID: CCFL; datasetName: Anticosti 1993
- **Type status:** Other material. **Occurrence:** recordNumber: 1993-3-4479; recordedBy: HEBECH01; individualCount: 54; sex: M; lifeStage: CI; preparations: pinned; disposition: in collection; **Taxon:** scientificNameID: CERASPONUPIF; family: Cerambycidae; taxonRank: Organism; scientificNameAuthorship: Neospondylis upiformis (Mannerheim 1843); **Location:** locationID: 1530; locality: Jupiter River; verbatimLatitude: 4931; verbatimLongitude: 6321; **Identification:** identifiedBy: FRECMA01; dateIdentified: 1997; **Event:** samplingProtocol: 12 funnel Lindgren traps, baited with 95% Ethanol and α-pinene; habitat: Old-growth balsam fir forest; **Record Level:** collectionID: CCFL; datasetName: Anticosti 1993
- **Type status:** Other material. **Occurrence:** recordNumber: 1993-3-4479; recordedBy: HEBECH01; individualCount: 69; sex: F; lifeStage: CI; preparations: pinned; disposition: in collection; **Taxon:** scientificNameID: CERASPONUPIF; family: Cerambycidae; taxonRank: Organism; scientificNameAuthorship: Neospondylis upiformis (Mannerheim 1843); **Location:** locationID: 1530; locality: Jupiter River; verbatimLatitude: 4931; verbatimLongitude: 6321; **Identification:** identifiedBy: FRECMA01; dateIdentified: 1997; **Event:** samplingProtocol: 12 funnel Lindgren traps, baited with 95% Ethanol and α-pinene; habitat: Old-growth balsam fir forest; **Record Level:** collectionID: CCFL; datasetName: Anticosti 1993
- **Type status:** Other material. **Occurrence:** recordNumber: 1993-3-4479; recordedBy: HEBECH01; individualCount: 4; sex: I; lifeStage: CI; disposition: missing; **Taxon:** scientificNameID: CERASPONUPIF; family: Cerambycidae; taxonRank: Organism; scientificNameAuthorship: Neospondylis upiformis (Mannerheim 1843); **Location:** locationID: 1530; locality: Jupiter River; verbatimLatitude: 4931; verbatimLongitude: 6321; **Identification:** identifiedBy: FRECMA01; dateIdentified: 1997; **Event:** samplingProtocol: 12 funnel Lindgren traps, baited with 95% Ethanol and α-pinene; habitat: Old-growth balsam fir forest; **Record Level:** datasetName: Anticosti 1993
- **Type status:** Other material. **Occurrence:** recordNumber: 1993-3-4484; recordedBy: HEBECH01; individualCount: 7; sex: M; lifeStage: CI; preparations: pinned; disposition: in collection; **Taxon:** scientificNameID: CERASPONUPIF; family: Cerambycidae; taxonRank: Organism; scientificNameAuthorship: Neospondylis upiformis (Mannerheim 1843); **Location:** locationID: 1530; locality: Jupiter River; verbatimLatitude: 4931; verbatimLongitude: 6321; **Identification:** identifiedBy: FRECMA01; dateIdentified: 1997; **Event:** samplingProtocol: 12 funnel Lindgren traps, baited with 95% Ethanol and α-pinene; habitat: Old-growth balsam fir forest; **Record Level:** collectionID: CCFL; datasetName: Anticosti 1993
- **Type status:** Other material. **Occurrence:** recordNumber: 1993-3-4484; recordedBy: HEBECH01; individualCount: 16; sex: F; lifeStage: CI; preparations: pinned; disposition: in collection; **Taxon:** scientificNameID: CERASPONUPIF; family: Cerambycidae; taxonRank: Organism; scientificNameAuthorship: Neospondylis upiformis (Mannerheim 1843); **Location:** locationID: 1530; locality: Jupiter River; verbatimLatitude: 4931; verbatimLongitude: 6321; **Identification:** identifiedBy: FRECMA01; dateIdentified: 1997; **Event:** samplingProtocol: 12 funnel Lindgren traps, baited with 95% Ethanol and α-pinene; habitat: Old-growth balsam fir forest; **Record Level:** collectionID: CCFL; datasetName: Anticosti 1993
- **Type status:** Other material. **Occurrence:** recordNumber: 1993-3-4494; recordedBy: HEBECH01; individualCount: 1; sex: M; lifeStage: CI; preparations: pinned; disposition: in collection; **Taxon:** scientificNameID: CERASPONUPIF; family: Cerambycidae; taxonRank: Organism; scientificNameAuthorship: Neospondylis upiformis (Mannerheim 1843); **Location:** locationID: 1530; locality: Jupiter River; verbatimLatitude: 4931; verbatimLongitude: 6321; **Identification:** identifiedBy: FRECMA01; dateIdentified: 1997; **Event:** samplingProtocol: 12 funnel Lindgren traps, baited with 95% Ethanol and α-pinene; habitat: Old-growth balsam fir forest; **Record Level:** collectionID: CCFL; datasetName: Anticosti 1993
- **Type status:** Other material. **Occurrence:** recordNumber: 1993-3-4494; recordedBy: HEBECH01; individualCount: 1; sex: F; lifeStage: CI; preparations: pinned; disposition: in collection; **Taxon:** scientificNameID: CERASPONUPIF; family: Cerambycidae; taxonRank: Organism; scientificNameAuthorship: Neospondylis upiformis (Mannerheim 1843); **Location:** locationID: 1530; locality: Jupiter River; verbatimLatitude: 4931; verbatimLongitude: 6321; **Identification:** identifiedBy: FRECMA01; dateIdentified: 1997; **Event:** samplingProtocol: 12 funnel Lindgren traps, baited with 95% Ethanol and α-pinene; habitat: Old-growth balsam fir forest; **Record Level:** collectionID: CCFL; datasetName: Anticosti 1993
- **Type status:** Other material. **Occurrence:** recordNumber: 1993-3-4554; recordedBy: HEBECH01; individualCount: 1; sex: F; lifeStage: CI; preparations: pinned; disposition: in collection; **Taxon:** scientificNameID: CERASPONUPIF; family: Cerambycidae; taxonRank: Organism; scientificNameAuthorship: Neospondylis upiformis (Mannerheim 1843); **Location:** locationID: 1530; locality: South-West Point; verbatimLatitude: 4927; verbatimLongitude: 6324; **Identification:** identifiedBy: FRECMA01; dateIdentified: 1997; **Event:** samplingProtocol: 12 funnel Lindgren traps, baited with 95% Ethanol and α-pinene; habitat: White spruce forest surrounded by old-growth balsam fir forest; **Record Level:** collectionID: CCFL; datasetName: Anticosti 1993
- **Type status:** Other material. **Occurrence:** recordNumber: 1993-3-4559; recordedBy: HEBECH01; individualCount: 4; sex: F; lifeStage: CI; preparations: pinned; disposition: in collection; **Taxon:** scientificNameID: CERASPONUPIF; family: Cerambycidae; taxonRank: Organism; scientificNameAuthorship: Neospondylis upiformis (Mannerheim 1843); **Location:** locationID: 1530; locality: South-West Point; verbatimLatitude: 4927; verbatimLongitude: 6324; **Identification:** identifiedBy: FRECMA01; dateIdentified: 1997; **Event:** samplingProtocol: 12 funnel Lindgren traps, baited with 95% Ethanol and α-pinene; habitat: White spruce forest surrounded by old-growth balsam fir forest; **Record Level:** collectionID: CCFL; datasetName: Anticosti 1993
- **Type status:** Other material. **Occurrence:** recordNumber: 1993-3-4569; recordedBy: HEBECH01; individualCount: 10; sex: M; lifeStage: CI; preparations: pinned; disposition: in collection; **Taxon:** scientificNameID: CERASPONUPIF; family: Cerambycidae; taxonRank: Organism; scientificNameAuthorship: Neospondylis upiformis (Mannerheim 1843); **Location:** locationID: 1530; locality: South-West Point; verbatimLatitude: 4927; verbatimLongitude: 6324; **Identification:** identifiedBy: FRECMA01; dateIdentified: 1997; **Event:** samplingProtocol: 12 funnel Lindgren traps, baited with 95% Ethanol and α-pinene; habitat: White spruce forest surrounded by old-growth balsam fir forest; **Record Level:** collectionID: CCFL; datasetName: Anticosti 1993
- **Type status:** Other material. **Occurrence:** recordNumber: 1993-3-4569; recordedBy: HEBECH01; individualCount: 5; sex: F; lifeStage: CI; preparations: pinned; disposition: in collection; **Taxon:** scientificNameID: CERASPONUPIF; family: Cerambycidae; taxonRank: Organism; scientificNameAuthorship: Neospondylis upiformis (Mannerheim 1843); **Location:** locationID: 1530; locality: South-West Point; verbatimLatitude: 4927; verbatimLongitude: 6324; **Identification:** identifiedBy: FRECMA01; dateIdentified: 1997; **Event:** samplingProtocol: 12 funnel Lindgren traps, baited with 95% Ethanol and α-pinene; habitat: White spruce forest surrounded by old-growth balsam fir forest; **Record Level:** collectionID: CCFL; datasetName: Anticosti 1993
- **Type status:** Other material. **Occurrence:** recordNumber: 1993-3-4569; recordedBy: HEBECH01; individualCount: 3; sex: I; lifeStage: CI; disposition: missing; **Taxon:** scientificNameID: CERASPONUPIF; family: Cerambycidae; taxonRank: Organism; scientificNameAuthorship: Neospondylis upiformis (Mannerheim 1843); **Location:** locationID: 1530; locality: South-West Point; verbatimLatitude: 4927; verbatimLongitude: 6324; **Identification:** identifiedBy: FRECMA01; dateIdentified: 1997; **Event:** samplingProtocol: 12 funnel Lindgren traps, baited with 95% Ethanol and α-pinene; habitat: White spruce forest surrounded by old-growth balsam fir forest; **Record Level:** datasetName: Anticosti 1993
- **Type status:** Other material. **Occurrence:** recordNumber: 1993-3-4574; recordedBy: HEBECH01; individualCount: 12; sex: M; lifeStage: CI; preparations: pinned; disposition: in collection; **Taxon:** scientificNameID: CERASPONUPIF; family: Cerambycidae; taxonRank: Organism; scientificNameAuthorship: Neospondylis upiformis (Mannerheim 1843); **Location:** locationID: 1530; locality: South-West Point; verbatimLatitude: 4927; verbatimLongitude: 6324; **Identification:** identifiedBy: FRECMA01; dateIdentified: 1997; **Event:** samplingProtocol: 12 funnel Lindgren traps, baited with 95% Ethanol and α-pinene; habitat: White spruce forest surrounded by old-growth balsam fir forest; **Record Level:** collectionID: CCFL; datasetName: Anticosti 1993
- **Type status:** Other material. **Occurrence:** recordNumber: 1993-3-4574; recordedBy: HEBECH01; individualCount: 16; sex: F; lifeStage: CI; preparations: pinned; disposition: in collection; **Taxon:** scientificNameID: CERASPONUPIF; family: Cerambycidae; taxonRank: Organism; scientificNameAuthorship: Neospondylis upiformis (Mannerheim 1843); **Location:** locationID: 1530; locality: South-West Point; verbatimLatitude: 4927; verbatimLongitude: 6324; **Identification:** identifiedBy: FRECMA01; dateIdentified: 1997; **Event:** samplingProtocol: 12 funnel Lindgren traps, baited with 95% Ethanol and α-pinene; habitat: White spruce forest surrounded by old-growth balsam fir forest; **Record Level:** collectionID: CCFL; datasetName: Anticosti 1993
- **Type status:** Other material. **Occurrence:** recordNumber: 1993-3-4619; recordedBy: HEBECH01; individualCount: 1; sex: M; lifeStage: CI; preparations: pinned; disposition: in collection; **Taxon:** scientificNameID: CERASPONUPIF; family: Cerambycidae; taxonRank: Organism; scientificNameAuthorship: Neospondylis upiformis (Mannerheim 1843); **Location:** locationID: 1530; locality: Macdonald River; verbatimLatitude: 4945; verbatimLongitude: 6305; **Identification:** identifiedBy: FRECMA01; dateIdentified: 1997; **Event:** samplingProtocol: 12 funnel Lindgren traps, baited with 95% Ethanol and α-pinene; habitat: Old-growth balsam fir forest; **Record Level:** collectionID: CCFL; datasetName: Anticosti 1993
- **Type status:** Other material. **Occurrence:** recordNumber: 1993-3-4619; recordedBy: HEBECH01; individualCount: 3; sex: F; lifeStage: CI; preparations: pinned; disposition: in collection; **Taxon:** scientificNameID: CERASPONUPIF; family: Cerambycidae; taxonRank: Organism; scientificNameAuthorship: Neospondylis upiformis (Mannerheim 1843); **Location:** locationID: 1530; locality: Macdonald River; verbatimLatitude: 4945; verbatimLongitude: 6305; **Identification:** identifiedBy: FRECMA01; dateIdentified: 1997; **Event:** samplingProtocol: 12 funnel Lindgren traps, baited with 95% Ethanol and α-pinene; habitat: Old-growth balsam fir forest; **Record Level:** collectionID: CCFL; datasetName: Anticosti 1993
- **Type status:** Other material. **Occurrence:** recordNumber: 1993-3-4624; recordedBy: HEBECH01; individualCount: 2; sex: M; lifeStage: CI; preparations: pinned; disposition: in collection; **Taxon:** scientificNameID: CERASPONUPIF; family: Cerambycidae; taxonRank: Organism; scientificNameAuthorship: Neospondylis upiformis (Mannerheim 1843); **Location:** locationID: 1530; locality: Macdonald River; verbatimLatitude: 4945; verbatimLongitude: 6305; **Identification:** identifiedBy: FRECMA01; dateIdentified: 1997; **Event:** samplingProtocol: 12 funnel Lindgren traps, baited with 95% Ethanol and α-pinene; habitat: Old-growth balsam fir forest; **Record Level:** collectionID: CCFL; datasetName: Anticosti 1993
- **Type status:** Other material. **Occurrence:** recordNumber: 1993-3-4624; recordedBy: HEBECH01; individualCount: 5; sex: F; lifeStage: CI; preparations: pinned; disposition: in collection; **Taxon:** scientificNameID: CERASPONUPIF; family: Cerambycidae; taxonRank: Organism; scientificNameAuthorship: Neospondylis upiformis (Mannerheim 1843); **Location:** locationID: 1530; locality: Macdonald River; verbatimLatitude: 4945; verbatimLongitude: 6305; **Identification:** identifiedBy: FRECMA01; dateIdentified: 1997; **Event:** samplingProtocol: 12 funnel Lindgren traps, baited with 95% Ethanol and α-pinene; habitat: Old-growth balsam fir forest; **Record Level:** collectionID: CCFL; datasetName: Anticosti 1993
- **Type status:** Other material. **Occurrence:** recordNumber: 1994-3-0025; recordedBy: HEBECH01; individualCount: 1; sex: I; lifeStage: CI; disposition: missing; **Taxon:** scientificNameID: CERASPONUPIF; family: Cerambycidae; taxonRank: Organism; scientificNameAuthorship: Neospondylis upiformis (Mannerheim 1843); **Location:** locality: Armagh; verbatimLatitude: 4645; verbatimLongitude: 7035; **Identification:** identifiedBy: FRECMA01; dateIdentified: 1995; **Event:** samplingProtocol: 12 funnel Lindgren traps, baited with 95% Ethanol and α-pinene; habitat: Mature balsam fir forest; **Record Level:** datasetName: Réseau sapiničre 1994
- **Type status:** Other material. **Occurrence:** recordNumber: 1994-3-0836; recordedBy: HEBECH01; individualCount: 2; sex: M; lifeStage: CI; preparations: pinned; disposition: in collection; **Taxon:** scientificNameID: CERASPONUPIF; family: Cerambycidae; taxonRank: Organism; scientificNameAuthorship: Neospondylis upiformis (Mannerheim 1843); **Location:** locationID: 2902; locality: Pellegrin; verbatimLatitude: 4832; verbatimLongitude: 6454; **Identification:** identifiedBy: FRECMA01; dateIdentified: 1997; **Event:** samplingProtocol: 12 funnel Lindgren traps, baited with 95% Ethanol and α-pinene; habitat: Mature balsam fir forest; **Record Level:** collectionID: CCFL; datasetName: Réseau sapiničre 1994
- **Type status:** Other material. **Occurrence:** recordNumber: 1994-3-0887; recordedBy: HEBECH01; individualCount: 1; sex: M; lifeStage: CI; preparations: pinned; disposition: in collection; **Taxon:** scientificNameID: CERASPONUPIF; family: Cerambycidae; taxonRank: Organism; scientificNameAuthorship: Neospondylis upiformis (Mannerheim 1843); **Location:** locationID: 13095; locality: Pohenegamook; verbatimLatitude: 4737; verbatimLongitude: 6917; **Identification:** identifiedBy: FRECMA01; dateIdentified: 1997; **Event:** samplingProtocol: 12 funnel Lindgren traps, baited with 95% Ethanol and α-pinene; habitat: Mature balsam fir forest; **Record Level:** collectionID: CCFL; datasetName: Réseau sapiničre 1994
- **Type status:** Other material. **Occurrence:** recordNumber: 1998-3-0932; recordedBy: HEBECH01; individualCount: 2; sex: F; lifeStage: CI; preparations: pinned; disposition: in collection; **Taxon:** scientificNameID: CERASPONUPIF; family: Cerambycidae; taxonRank: Organism; scientificNameAuthorship: Neospondylis upiformis (Mannerheim 1843); **Location:** locationID: 1530; locality: Jupiter River; verbatimLatitude: 4932; verbatimLongitude: 6323; **Identification:** identifiedBy: DUBUYV01; dateIdentified: 2006; **Event:** samplingProtocol: 12 funnel Lindgren traps, baited with 95% Ethanol and α-pinene; habitat: Old-growth balsam fir forest; **Record Level:** collectionID: CCFL; datasetName: Tests Lindgren
- **Type status:** Other material. **Occurrence:** recordNumber: 1998-3-0933; recordedBy: HEBECH01; individualCount: 1; sex: M; lifeStage: CI; preparations: pinned; disposition: in collection; **Taxon:** scientificNameID: CERASPONUPIF; family: Cerambycidae; taxonRank: Organism; scientificNameAuthorship: Neospondylis upiformis (Mannerheim 1843); **Location:** locationID: 1530; locality: Jupiter River; verbatimLatitude: 4932; verbatimLongitude: 6323; **Identification:** identifiedBy: DUBUYV01; dateIdentified: 2006; **Event:** samplingProtocol: 12 funnel Lindgren traps, baited with 95% Ethanol and α-pinene; habitat: Old-growth balsam fir forest; **Record Level:** collectionID: CCFL; datasetName: Tests Lindgren
- **Type status:** Other material. **Occurrence:** recordNumber: 1998-3-0936; recordedBy: HEBECH01; individualCount: 1; sex: F; lifeStage: CI; preparations: pinned; disposition: in collection; **Taxon:** scientificNameID: CERASPONUPIF; family: Cerambycidae; taxonRank: Organism; scientificNameAuthorship: Neospondylis upiformis (Mannerheim 1843); **Location:** locationID: 1530; locality: Jupiter River; verbatimLatitude: 4932; verbatimLongitude: 6221; **Identification:** identifiedBy: DUBUYV01; dateIdentified: 2006; **Event:** samplingProtocol: 12 funnel Lindgren traps, baited with 95% Ethanol and α-pinene; habitat: Old-growth balsam fir forest; **Record Level:** collectionID: CCFL; datasetName: Tests Lindgren
- **Type status:** Other material. **Occurrence:** occurrenceRemarks: Barcode of life, Sample ID LFCa-08-113; recordNumber: 2001-3-3959; recordedBy: HEBECH01; individualCount: 1; sex: M; lifeStage: CI; preparations: pinned; disposition: in collection; **Taxon:** scientificNameID: CERASPONUPIF; family: Cerambycidae; taxonRank: Organism; scientificNameAuthorship: Neospondylis upiformis (Mannerheim 1843); **Location:** locationID: 43027; locality: Huntingville; verbatimLatitude: 4519; verbatimLongitude: 7149; **Identification:** identifiedBy: PELLGE01; dateIdentified: 2004; **Event:** samplingProtocol: 12 funnel Lindgren traps, baited with 95% Ethanol and α-pinene; habitat: Red pine plantation; **Record Level:** collectionID: CCFL; datasetName: Grand Hylésine
- **Type status:** Other material. **Occurrence:** recordNumber: 2007-3-3534; recordedBy: HEBECH01; individualCount: 2; sex: M; lifeStage: CI; preparations: pinned; disposition: in collection; **Taxon:** scientificNameID: CERASPONUPIF; family: Cerambycidae; taxonRank: Organism; scientificNameAuthorship: Neospondylis upiformis (Mannerheim 1843); **Location:** locationID: 1530; locality: Lac McRay; verbatimLatitude: 4952; verbatimLongitude: 6404; **Identification:** identifiedBy: DUBUYV01; dateIdentified: 2007; **Event:** samplingProtocol: 12 funnel Lindgren traps, baited with 95% Ethanol and α-pinene; habitat: Mature balsam fir forest; **Record Level:** collectionID: CCFL; datasetName: Anticosti 2007
- **Type status:** Other material. **Occurrence:** recordNumber: 2007-3-3534; recordedBy: HEBECH01; individualCount: 1; sex: F; lifeStage: CI; preparations: pinned; disposition: in collection; **Taxon:** scientificNameID: CERASPONUPIF; family: Cerambycidae; taxonRank: Organism; scientificNameAuthorship: Neospondylis upiformis (Mannerheim 1843); **Location:** locationID: 1530; locality: Lac McRay; verbatimLatitude: 4952; verbatimLongitude: 6404; **Identification:** identifiedBy: DUBUYV01; dateIdentified: 2007; **Event:** samplingProtocol: 12 funnel Lindgren traps, baited with 95% Ethanol and α-pinene; habitat: Mature balsam fir forest; **Record Level:** collectionID: CCFL; datasetName: Anticosti 2007
- **Type status:** Other material. **Occurrence:** recordNumber: 2007-3-3535; recordedBy: HEBECH01; individualCount: 1; sex: M; lifeStage: CI; preparations: pinned; disposition: in collection; **Taxon:** scientificNameID: CERASPONUPIF; family: Cerambycidae; taxonRank: Organism; scientificNameAuthorship: Neospondylis upiformis (Mannerheim 1843); **Location:** locationID: 1530; locality: Rivičre Jupiter; verbatimLatitude: 4932; verbatimLongitude: 6318; **Identification:** identifiedBy: DUBUYV01; dateIdentified: 2007; **Event:** samplingProtocol: 12 funnel Lindgren traps, baited with 95% Ethanol and α-pinene; habitat: Old-growth balsam fir forest; **Record Level:** collectionID: CCFL; datasetName: Anticosti 2007
- **Type status:** Other material. **Occurrence:** recordNumber: 2007-3-3535; recordedBy: HEBECH01; individualCount: 1; sex: F; lifeStage: CI; preparations: pinned; disposition: in collection; **Taxon:** scientificNameID: CERASPONUPIF; family: Cerambycidae; taxonRank: Organism; scientificNameAuthorship: Neospondylis upiformis (Mannerheim 1843); **Location:** locationID: 1530; locality: Rivičre Jupiter; verbatimLatitude: 4932; verbatimLongitude: 6318; **Identification:** identifiedBy: DUBUYV01; dateIdentified: 2007; **Event:** samplingProtocol: 12 funnel Lindgren traps, baited with 95% Ethanol and α-pinene; habitat: Old-growth balsam fir forest; **Record Level:** collectionID: CCFL; datasetName: Anticosti 2007
- **Type status:** Other material. **Occurrence:** recordNumber: 2007-3-3536; recordedBy: HEBECH01; individualCount: 12; sex: M; lifeStage: CI; preparations: pinned; disposition: in collection; **Taxon:** scientificNameID: CERASPONUPIF; family: Cerambycidae; taxonRank: Organism; scientificNameAuthorship: Neospondylis upiformis (Mannerheim 1843); **Location:** locationID: 1530; locality: Rivičre Jupiter; verbatimLatitude: 4931; verbatimLongitude: 6320; **Identification:** identifiedBy: DUBUYV01; dateIdentified: 2007; **Event:** samplingProtocol: 12 funnel Lindgren traps, baited with 95% Ethanol and α-pinene; habitat: Old-growth balsam fir forest; **Record Level:** collectionID: CCFL; datasetName: Anticosti 2007
- **Type status:** Other material. **Occurrence:** recordNumber: 2007-3-3536; recordedBy: HEBECH01; individualCount: 6; sex: F; lifeStage: CI; preparations: pinned; disposition: in collection; **Taxon:** scientificNameID: CERASPONUPIF; family: Cerambycidae; taxonRank: Organism; scientificNameAuthorship: Neospondylis upiformis (Mannerheim 1843); **Location:** locationID: 1530; locality: Rivičre Jupiter; verbatimLatitude: 4931; verbatimLongitude: 6320; **Identification:** identifiedBy: DUBUYV01; dateIdentified: 2007; **Event:** samplingProtocol: 12 funnel Lindgren traps, baited with 95% Ethanol and α-pinene; habitat: Old-growth balsam fir forest; **Record Level:** collectionID: CCFL; datasetName: Anticosti 2007
- **Type status:** Other material. **Occurrence:** recordNumber: 2007-3-3537; recordedBy: HEBECH01; individualCount: 6; sex: M; lifeStage: CI; preparations: pinned; disposition: in collection; **Taxon:** scientificNameID: CERASPONUPIF; family: Cerambycidae; taxonRank: Organism; scientificNameAuthorship: Neospondylis upiformis (Mannerheim 1843); **Location:** locationID: 1530; locality: Rivičre Jupiter; verbatimLatitude: 4931; verbatimLongitude: 6320; **Identification:** identifiedBy: DUBUYV01; dateIdentified: 2007; **Event:** samplingProtocol: 12 funnel Lindgren traps, baited with 95% Ethanol and α-pinene; habitat: Old-growth balsam fir forest; **Record Level:** collectionID: CCFL; datasetName: Anticosti 2007
- **Type status:** Other material. **Occurrence:** recordNumber: 2007-3-3537; recordedBy: HEBECH01; individualCount: 7; sex: F; lifeStage: CI; preparations: pinned; disposition: in collection; **Taxon:** scientificNameID: CERASPONUPIF; family: Cerambycidae; taxonRank: Organism; scientificNameAuthorship: Neospondylis upiformis (Mannerheim 1843); **Location:** locationID: 1530; locality: Rivičre Jupiter; verbatimLatitude: 4931; verbatimLongitude: 6320; **Identification:** identifiedBy: DUBUYV01; dateIdentified: 2007; **Event:** samplingProtocol: 12 funnel Lindgren traps, baited with 95% Ethanol and α-pinene; habitat: Old-growth balsam fir forest; **Record Level:** collectionID: CCFL; datasetName: Anticosti 2007
- **Type status:** Other material. **Occurrence:** recordNumber: 2007-3-3538; recordedBy: HEBECH01; individualCount: 2; sex: M; lifeStage: CI; preparations: pinned; disposition: in collection; **Taxon:** scientificNameID: CERASPONUPIF; family: Cerambycidae; taxonRank: Organism; scientificNameAuthorship: Neospondylis upiformis (Mannerheim 1843); **Location:** locationID: 1530; locality: Rivičre Jupiter; verbatimLatitude: 4932; verbatimLongitude: 6320; **Identification:** identifiedBy: DUBUYV01; dateIdentified: 2007; **Event:** samplingProtocol: 12 funnel Lindgren traps, baited with 95% Ethanol and α-pinene; habitat: Old-growth balsam fir forest; **Record Level:** collectionID: CCFL; datasetName: Anticosti 2007
- **Type status:** Other material. **Occurrence:** recordNumber: 2007-3-3538; recordedBy: HEBECH01; individualCount: 1; sex: F; lifeStage: CI; preparations: pinned; disposition: in collection; **Taxon:** scientificNameID: CERASPONUPIF; family: Cerambycidae; taxonRank: Organism; scientificNameAuthorship: Neospondylis upiformis (Mannerheim 1843); **Location:** locationID: 1530; locality: Rivičre Jupiter; verbatimLatitude: 4932; verbatimLongitude: 6320; **Identification:** identifiedBy: DUBUYV01; dateIdentified: 2007; **Event:** samplingProtocol: 12 funnel Lindgren traps, baited with 95% Ethanol and α-pinene; habitat: Old-growth balsam fir forest; **Record Level:** collectionID: CCFL; datasetName: Anticosti 2007
- **Type status:** Other material. **Occurrence:** recordNumber: 2007-3-3541; recordedBy: HEBECH01; individualCount: 2; sex: M; lifeStage: CI; preparations: pinned; disposition: in collection; **Taxon:** scientificNameID: CERASPONUPIF; family: Cerambycidae; taxonRank: Organism; scientificNameAuthorship: Neospondylis upiformis (Mannerheim 1843); **Location:** locationID: 1530; locality: Rivičre Jupiter; verbatimLatitude: 4932; verbatimLongitude: 6318; **Identification:** identifiedBy: DUBUYV01; dateIdentified: 2007; **Event:** samplingProtocol: 12 funnel Lindgren traps, baited with 95% Ethanol and α-pinene; habitat: Old-growth balsam fir forest; **Record Level:** collectionID: CCFL; datasetName: Anticosti 2007
- **Type status:** Other material. **Occurrence:** recordNumber: 2007-3-3541; recordedBy: HEBECH01; individualCount: 2; sex: F; lifeStage: CI; preparations: pinned; disposition: in collection; **Taxon:** scientificNameID: CERASPONUPIF; family: Cerambycidae; taxonRank: Organism; scientificNameAuthorship: Neospondylis upiformis (Mannerheim 1843); **Location:** locationID: 1530; locality: Rivičre Jupiter; verbatimLatitude: 4932; verbatimLongitude: 6318; **Identification:** identifiedBy: DUBUYV01; dateIdentified: 2007; **Event:** samplingProtocol: 12 funnel Lindgren traps, baited with 95% Ethanol and α-pinene; habitat: Old-growth balsam fir forest; **Record Level:** collectionID: CCFL; datasetName: Anticosti 2007
- **Type status:** Other material. **Occurrence:** recordNumber: 2007-3-3542; recordedBy: HEBECH01; individualCount: 6; sex: M; lifeStage: CI; preparations: pinned; disposition: in collection; **Taxon:** scientificNameID: CERASPONUPIF; family: Cerambycidae; taxonRank: Organism; scientificNameAuthorship: Neospondylis upiformis (Mannerheim 1843); **Location:** locationID: 1530; locality: Rivičre Jupiter; verbatimLatitude: 4931; verbatimLongitude: 6320; **Identification:** identifiedBy: DUBUYV01; dateIdentified: 2007; **Event:** samplingProtocol: 12 funnel Lindgren traps, baited with 95% Ethanol and α-pinene; habitat: Old-growth balsam fir forest; **Record Level:** collectionID: CCFL; datasetName: Anticosti 2007
- **Type status:** Other material. **Occurrence:** recordNumber: 2007-3-3542; recordedBy: HEBECH01; individualCount: 7; sex: F; lifeStage: CI; preparations: pinned; disposition: in collection; **Taxon:** scientificNameID: CERASPONUPIF; family: Cerambycidae; taxonRank: Organism; scientificNameAuthorship: Neospondylis upiformis (Mannerheim 1843); **Location:** locationID: 1530; locality: Rivičre Jupiter; verbatimLatitude: 4931; verbatimLongitude: 6320; **Identification:** identifiedBy: DUBUYV01; dateIdentified: 2007; **Event:** samplingProtocol: 12 funnel Lindgren traps, baited with 95% Ethanol and α-pinene; habitat: Old-growth balsam fir forest; **Record Level:** collectionID: CCFL; datasetName: Anticosti 2007
- **Type status:** Other material. **Occurrence:** recordNumber: 2007-3-3543; recordedBy: HEBECH01; individualCount: 4; sex: F; lifeStage: CI; preparations: pinned; disposition: in collection; **Taxon:** scientificNameID: CERASPONUPIF; family: Cerambycidae; taxonRank: Organism; scientificNameAuthorship: Neospondylis upiformis (Mannerheim 1843); **Location:** locationID: 1530; locality: Rivičre Jupiter; verbatimLatitude: 4931; verbatimLongitude: 6320; **Identification:** identifiedBy: DUBUYV01; dateIdentified: 2007; **Event:** samplingProtocol: 12 funnel Lindgren traps, baited with 95% Ethanol and α-pinene; habitat: Old-growth balsam fir forest; **Record Level:** collectionID: CCFL; datasetName: Anticosti 2007
- **Type status:** Other material. **Occurrence:** recordNumber: 2007-3-3543; recordedBy: HEBECH01; individualCount: 1; sex: M; lifeStage: CI; preparations: pinned; disposition: in collection; **Taxon:** scientificNameID: CERASPONUPIF; family: Cerambycidae; taxonRank: Organism; scientificNameAuthorship: Neospondylis upiformis (Mannerheim 1843); **Location:** locationID: 1530; locality: Rivičre Jupiter; verbatimLatitude: 4931; verbatimLongitude: 6320; **Identification:** identifiedBy: DUBUYV01; dateIdentified: 2007; **Event:** samplingProtocol: 12 funnel Lindgren traps, baited with 95% Ethanol and α-pinene; habitat: Old-growth balsam fir forest; **Record Level:** collectionID: CCFL; datasetName: Anticosti 2007
- **Type status:** Other material. **Occurrence:** recordNumber: 2007-3-3544; recordedBy: HEBECH01; individualCount: 1; sex: F; lifeStage: CI; preparations: pinned; disposition: in collection; **Taxon:** scientificNameID: CERASPONUPIF; family: Cerambycidae; taxonRank: Organism; scientificNameAuthorship: Neospondylis upiformis (Mannerheim 1843); **Location:** locationID: 1530; locality: Rivičre Jupiter; verbatimLatitude: 4932; verbatimLongitude: 6320; **Identification:** identifiedBy: DUBUYV01; dateIdentified: 2007; **Event:** samplingProtocol: 12 funnel Lindgren traps, baited with 95% Ethanol and α-pinene; habitat: Old-growth balsam fir forest; **Record Level:** collectionID: CCFL; datasetName: Anticosti 2007
- **Type status:** Other material. **Occurrence:** recordNumber: 2007-3-3546; recordedBy: HEBECH01; individualCount: 1; sex: F; lifeStage: CI; preparations: pinned; disposition: in collection; **Taxon:** scientificNameID: CERASPONUPIF; family: Cerambycidae; taxonRank: Organism; scientificNameAuthorship: Neospondylis upiformis (Mannerheim 1843); **Location:** locationID: 1530; locality: Lac McRay; verbatimLatitude: 4952; verbatimLongitude: 6404; **Identification:** identifiedBy: DUBUYV01; dateIdentified: 2007; **Event:** samplingProtocol: 12 funnel Lindgren traps, baited with 95% Ethanol and α-pinene; habitat: Mature balsam fir forest; **Record Level:** collectionID: CCFL; datasetName: Anticosti 2007
- **Type status:** Other material. **Occurrence:** recordNumber: 2007-3-3547; recordedBy: HEBECH01; individualCount: 1; sex: M; lifeStage: CI; preparations: pinned; disposition: in collection; **Taxon:** scientificNameID: CERASPONUPIF; family: Cerambycidae; taxonRank: Organism; scientificNameAuthorship: Neospondylis upiformis (Mannerheim 1843); **Location:** locationID: 1530; locality: Rivičre Jupiter; verbatimLatitude: 4932; verbatimLongitude: 6318; **Identification:** identifiedBy: DUBUYV01; dateIdentified: 2007; **Event:** samplingProtocol: 12 funnel Lindgren traps, baited with 95% Ethanol and α-pinene; habitat: Old-growth balsam fir forest; **Record Level:** collectionID: CCFL; datasetName: Anticosti 2007
- **Type status:** Other material. **Occurrence:** recordNumber: 2007-3-3547; recordedBy: HEBECH01; individualCount: 5; sex: F; lifeStage: CI; preparations: pinned; disposition: in collection; **Taxon:** scientificNameID: CERASPONUPIF; family: Cerambycidae; taxonRank: Organism; scientificNameAuthorship: Neospondylis upiformis (Mannerheim 1843); **Location:** locationID: 1530; locality: Rivičre Jupiter; verbatimLatitude: 4932; verbatimLongitude: 6318; **Identification:** identifiedBy: DUBUYV01; dateIdentified: 2007; **Event:** samplingProtocol: 12 funnel Lindgren traps, baited with 95% Ethanol and α-pinene; habitat: Old-growth balsam fir forest; **Record Level:** collectionID: CCFL; datasetName: Anticosti 2007
- **Type status:** Other material. **Occurrence:** occurrenceRemarks: Barcode of life, Sample ID LFCa-08-809; recordNumber: 2007-3-3548; recordedBy: HEBECH01; individualCount: 1; sex: M; lifeStage: CI; preparations: pinned; disposition: in collection; **Taxon:** scientificNameID: CERASPONUPIF; family: Cerambycidae; taxonRank: Organism; scientificNameAuthorship: Neospondylis upiformis (Mannerheim 1843); **Location:** locationID: 1530; locality: Rivičre Jupiter; verbatimLatitude: 4931; verbatimLongitude: 6320; **Identification:** identifiedBy: DUBUYV01; dateIdentified: 2007; **Event:** samplingProtocol: 12 funnel Lindgren traps, baited with 95% Ethanol and α-pinene; habitat: Old-growth balsam fir forest; **Record Level:** collectionID: CCFL; datasetName: Anticosti 2007
- **Type status:** Other material. **Occurrence:** occurrenceRemarks: Barcode of life, Sample ID LFCa-08-808; recordNumber: 2007-3-3548; recordedBy: HEBECH01; individualCount: 1; sex: F; lifeStage: CI; preparations: pinned; disposition: in collection; **Taxon:** scientificNameID: CERASPONUPIF; family: Cerambycidae; taxonRank: Organism; scientificNameAuthorship: Neospondylis upiformis (Mannerheim 1843); **Location:** locationID: 1530; locality: Rivičre Jupiter; verbatimLatitude: 4931; verbatimLongitude: 6320; **Identification:** identifiedBy: DUBUYV01; dateIdentified: 2007; **Event:** samplingProtocol: 12 funnel Lindgren traps, baited with 95% Ethanol and α-pinene; habitat: Old-growth balsam fir forest; **Record Level:** collectionID: CCFL; datasetName: Anticosti 2007
- **Type status:** Other material. **Occurrence:** occurrenceRemarks: Barcode of life, Sample ID LFCa-08-807; recordNumber: 2007-3-3549; recordedBy: HEBECH01; individualCount: 1; sex: M; lifeStage: CI; preparations: pinned; disposition: in collection; **Taxon:** scientificNameID: CERASPONUPIF; family: Cerambycidae; taxonRank: Organism; scientificNameAuthorship: Neospondylis upiformis (Mannerheim 1843); **Location:** locationID: 1530; locality: Rivičre Jupiter; verbatimLatitude: 4931; verbatimLongitude: 6320; **Identification:** identifiedBy: DUBUYV01; dateIdentified: 2007; **Event:** samplingProtocol: 12 funnel Lindgren traps, baited with 95% Ethanol and α-pinene; habitat: Old-growth balsam fir forest; **Record Level:** collectionID: CCFL; datasetName: Anticosti 2007

#### Taxon discussion

*Neospondylis upiformis* (Mannerheim) (Coleoptera: Cerambycidae, Spondylidinae) is a species formerly included in the genus *Spondylis* (Sama 2005).

## Analysis

**Figure 2.**
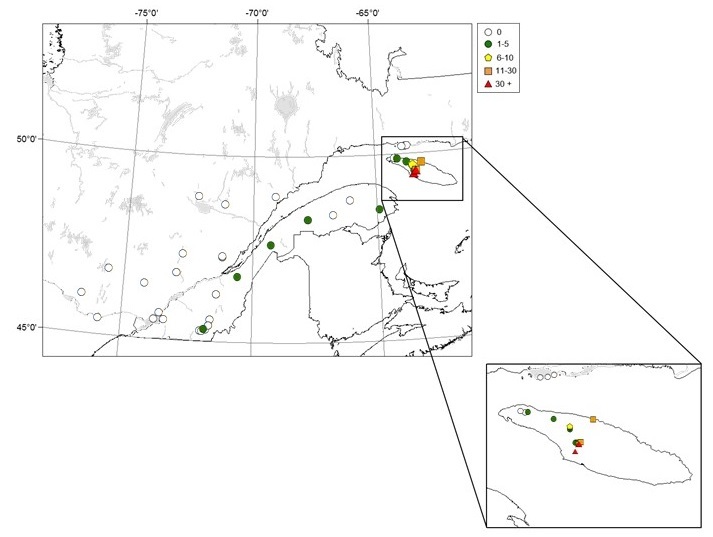
Distribution and abundance (N/trap) of *Neospondylis upiformis* caught in 12-funnel Lindgren traps, baited with 95% ethanol and α-pinene in the province of Quebec, Canada.

**Figure 3.**
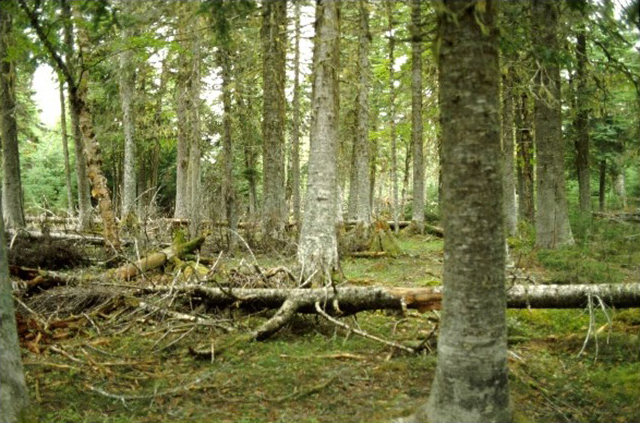
Old-growth forest of the Jupiter River area on Anticosti Island, Quebec, Canada.

A total of 340 *N. upiformis* adults were captured in baited Lindgren traps between 1993 and 2015 in 10 projects accounting for a total sampling effort of 5518 trap-days in the province of Quebec. It is many more than the 14 specimens found in 45 insect collections throughout the province. However, 333 of the 340 *N. upiformis* were collected on Anticosti Island, a location that had never been sampled before, which represents nearly 98% of the specimens captured for less than 24% of the sampling effort in our projects (Table 1). Adult *N. upiformis* were captured in seven out of eight forest stands sampled on Anticosti Island in 1993, attesting to the widespread presence of this uncommon species on the island (Table 1). The highest numbers of captures were recorded in the two sites located in the south-central part of the Island (Fig. 2), one old-growth balsam fir (*Abies balsamea* (L.) Mill.) stand (Riv. Jupiter) (Fig. 3) and one small white spruce stand (Pointe Sud-Ouest) surrounded by the same old-growth balsam fir forest matrix.

**Figure 4.**
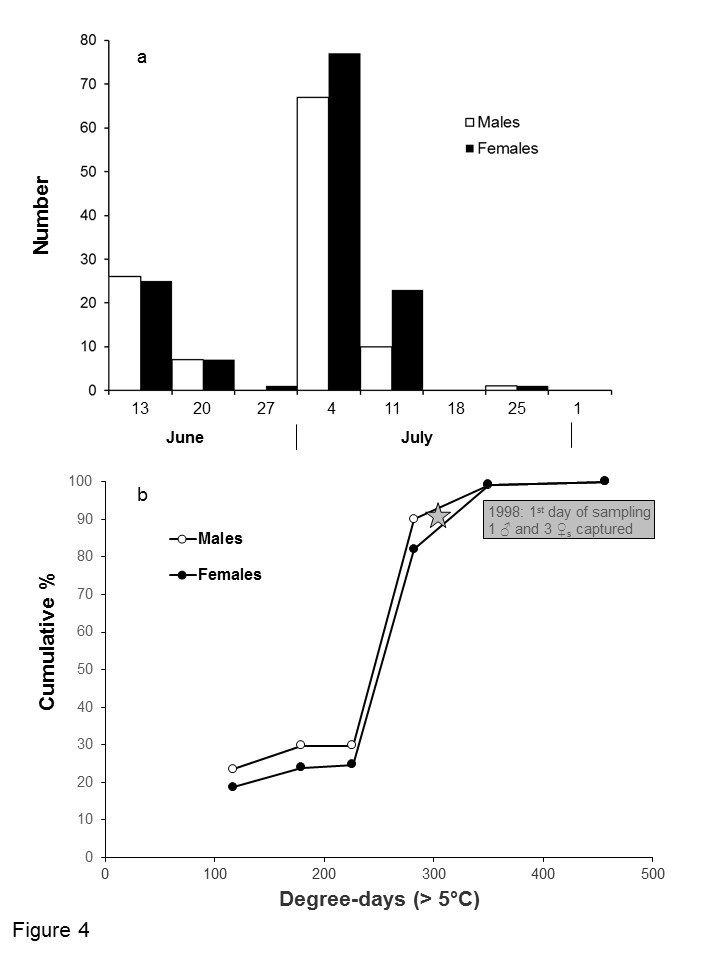
Weekly captures of males and females of *Neospondylis upiformis* in 12-funnel Lindgren traps baited with 95% ethanol and α-pinene on Anticosti Island, Quebec, Canada, in 1993.

The first beetles were caught at the beginning of the sampling period, between 9 and 17 June 1993 (Fig. 4). Captures then decreased, particularly in the last week of June when only one beetle was captured. However, a second peak was observed in early July, with three times more beetles being caught. Male seasonal captures were slightly earlier than those of females. No *N. upiformis* was caught after an accumulation of 350 degree-days above 5°C (Fig. 4).

In 1998, only four specimens were captured in a total of six traps located in the Jupiter River area (Table 1). These specimens were caught during the first week of sampling (29 June to 8 July). The 1998 season was much warmer than in 1993 and the 350 degrees-days threshold had already been reached on 1 July 1998, two days after the beginning of sampling (Fig. 4). This explains the low number of *N. upiformis* caught in 1998. Nevertheless, *N. upiformis* was still present and active in the same area where it had been so abundant 5 years earlier. In 2007, 68 more specimens were captured in four traps deployed in the Jupiter River area (Table 1; Fig. 2). On a trap-day basis, this was far less than in 1993 (2.42 vs 0.4 specimens/trap-day) but still, we caught 17 times more specimens in the Jupiter River area than in the western part of the island where we only caught four specimens in two traps placed near Lac McCrae (Table 1; Fig. 2).

Seven specimens were collected in five locations between 1993 and 2001, all on the south shore of the St. Lawrence River and in the eastern part of the province (Fig. 2). Six specimens were captured in four balsam fir stands in 1993 and 1994 (Table 1). In 1994, *N. upiformis* was not captured in the only balsam fir stand sampled on Anticosti Island, but it was located in the western part of the island. Additionally, *N. upiformis* was not found in the three balsam fir stands sampled on Mingan Islands in 2005 located on the north shore of the St. Lawrence River. The last specimen was caught in 2001 in a red pine plantation located in Huntingville, about 10 km south of Sherbrooke (Table 1; Fig. 2), during general surveys conducted to detect exotic insects.

**Figure 5.**
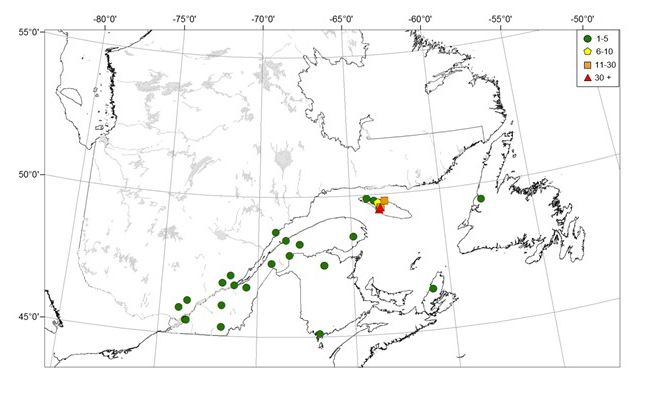
Map showing all the locations where *Neospondylis upiformis* was found in eastern Canada, with relative abundance at each location (Nb/location).

Overall, 14 *N. upiformis* specimens were found amongst 45 insect collections (Table 2). The earliest record dates back to 1925, while the most recent one was collected in 1964. Eight of the 14 records were from the Quebec City region and two were from the Island of Montreal (Table 2). Five records were from the north shore of the St. Lawrence River (Fig. 5). The furthest eastwards records were from Forestville and Rimouski, located respectively on the north and south shores of the St. Lawrence River. Apart from a specimen collected in June 1951, all specimens found in collections were collected in July and early August. Amongst the 12 specimens for which we had exact collection dates, eight were collected after 15 July (Table 2).

## Discussion

The data presented in this paper confirm that *N. upiformis* is uncommon in eastern Canada and has a very local distribution, as observed by Majka and Ogden (2010). However, our trapping results also suggest that Anticosti Island might be a hot spot for *N. upiformis* in eastern North America. We caught 333 specimens on this large island, which is far more than any other report from other regions of Quebec (7 specimens from 5 locations; this study), Newfoundland (4 specimens; Smith and Hurley 2005), New Brunswick (17 specimens; 5 in Webster et al. 2012; 4 as indicated by Webster pers. comm. 2018; 5 as indicated by Sweeney pers. comm. 2018) and Nova Scotia (12 specimens; Majka and Ogden 2010). This is also many more than the 14 specimens found in 45 insect collections in Quebec. Strangely, most specimens (8 out of 12 for which collection dates were available) found in collections were collected after 15 July while nearly all specimens collected on Anticosti Island were collected before that date, even if it is a much cooler area. Moreover, all specimens reported from Newfoundland (Smith and Hurley 2005), New Brunswick (Webster et al. 2012; Webster pers. comm. 2018; Sweeney pers. comm. 2018) and Nova Scotia (Majka and Ogden 2010) were collected before 15 July.

No records of *N. upiformis* had been reported by naturalists since 1964, suggesting that the habitat of this longhorned beetle may have rarified in southern Quebec. We did not capture any *N. upiformis* on the north shore of the St. Lawrence River, while five of the 14 specimens found in the collections were from this area. In fact, we only caught seven specimens elsewhere in the province of Quebec with attractive traps and a huge sampling effort over 22 years. The south-central part of Anticosti Island, to which belong the Jupiter River and the Pointe Sud-Ouest areas, was particularly rich in *N. upiformis* with 300 specimens out of the 333 caught on the island. These areas are mostly covered by old-growth balsam fir forests that survived previous hemlock looper, *Lambdina fiscellaria* (Guenée) (Lepidoptera: Geometridae), outbreaks (Jobin and Desaulniers 1981). The Jupiter River area was protected from extensive tree mortality by aerial spraying of Fenitrothion in the beginning of the 1970s (Jobin and Desaulniers 1981), but the mid-1930s outbreak was not controlled. Hemlock looper outbreaks usually cause higher mortality rates in smaller trees than in larger ones (MacLean and Ebert 1999). Such a mortality pattern should result in a more or less important natural thinning of balsam fir stands, favouring the growth of residual trees. This may explain the low density of large diameter balsam firs in the Jupiter River area (see Fig. 3). Larger trees should have larger roots as root biomass is closely linked with tree diameter (Bolte et al. 2004). This might be crucial for a species such as *N. upiformis* whose larvae feed and develop in roots (Gardiner 1970). The great abundance of *N. upiformis* in the south-central part of Anticosti Island might be linked to such particular conditions found in old-growth balsam fir forest.

The presence of *N. upiformis* has been reported in old-growth balsam fir and white spruce forests in protected areas of Newfoundland (Smith and Hurley 2005) and New Brunswick (Webster et al. 2012). In our study, the only stands where *N. upiformis* was not found on Anticosti Island were a white spruce stand (Lac Anna in 1993) and a balsam fir stand (Lac Princeton in 1994) located in the western part of the island where logging activities took place between 1910 and 1931 (Unpublished Reports and Maps). On the opposite, forests of the south-central part of the island had never been harvested before 2000. The landscape of the western part of the island is covered by “younger” (younger on Anticosti Island could be interpreted as overmature in the rest of the province as these forests are 60-90 years old) forests in which windthrow and natural senescence are less frequent than in the old-growth forests of the south-central part of the Island. This may partly explain the absence or rarity of *N. upiformis* in this part of the island as this species is known to take advantage of physiologically stressed trees (Goheen et al. 1985). The probability that physiologically stressed trees may occur is much higher in old-growth forests that are characterised by a higher rate of dead tree recruitment (Sippola et al. 1998). McCorquodale et al. (2007) suggested that the loss of old-growth forests may have played a role in the decline of longhorned beetles after 1950 in Ontario. Old-growth stands of the Jupiter River area have been partly harvested since 2000. Whether this might explain or not the decrease in abundance between 1993 and 2007 remains to be determined.

Whether Anticosti Island is a suitable habitat for *N. upiformis* because of the abundance of old-growth balsam fir forests, the presence of large white spruces, the particular climatic conditions or for any other reason is still unknown. White spruce is an increasing resource on the island and it should not limit *N. upiformis* abundance in the future, but old-growth balsam fir forests are rapidly disappearing (Potvin et al. 2003). Elsewhere in the province of Quebec, old-growth balsam fir forests have become rare due to logging (Desponts et al. 2002). It is interesting to note that 12 of the 14 early records (1925 to 1964) of *N. upiformis* were from western Quebec, even from the island of Montreal, where fir spruce forests were obviously older and more abundant in the first half of the 20th century than they are now. Activities of amateur entomologists have been intensive over the last 50 years in western and central Quebec. The absence of recent records of *N. upiformis* in these parts of the province suggests that fir spruce forest conditions have changed.

We captured *N. upiformis* efficiently with 12-funnel Lindgren traps baited with high release rate lures of 95% ethanol and α-pinene, the same baits used by Smith and Hurley (2005) in Newfoundland and by Webster et al. (2012) in New Brunswick. This trap and these lures are widely used in domestic surveys aimed at detecting exotic bark and woodboring beetles (Smith and Hurley 2005). The same lures have been used successfully with other types of traps to capture *Spondylis buprestoides*, a closely related European species (Shibata et al. 1996; Sweeney et al. 2004). Thus, the Lindgren multiple-funnel trap, baited with 95% ethanol and α-pinene, might also be efficient to capture *S. buprestoides*. This tool should improve the monitoring of *N. upiformis* in eastern Canada, but research is also needed to better define its habitat and improve knowledge on its biology and ecology; this is important for defining management strategies to maintain the populations of this species.

## Supplementary Material

Supplementary material 1 Cumulative degree-days above 5°C at Port-Menier on Anticosti Island in 2007 and comparison with data from Havre Saint-Pierre and Cap-des-Rosiers, respectively on the north and south shores of the St. Lawrence River or an average of these locations Data type: Graph
Brief description: Shows how seasonal cumulative degree-days on Anticosti Island follow that of Havre Saint-Pierre in May and of an average of Havre Saint-Pierre and Cap-des-Rosiers in June and July. Allows using proxies for 1993 and 1998 as we do not have complete data for Anticosti in these years.
File: oo_193115.pptx Christian Hébert

XML Treatment for Neospondylis upiformis

## Figures and Tables

**Table 1.** Sampling site locations, dominant tree species, sampling periods, trapping effort and abundance of *Neospondylis upiformis* caught in 12-funnel Lindgren traps baited with 95% ethanol and α-pinene in different research projects carried out between 1993 and 2015 in Quebec.

**Year**	**Project**	**Site**	**Long; Lat**	**Tree species**	**Sampling period**	**Nb traps**	**Trap x Days**	**Nb**	**Nb/TD**
1993	Anticosti	Lac Anna	64°07'W; 49°52'N	White spruce	Jun. 8 – Aug. 20	1	73	0	0
		Riv. Loutre	63°41'W; 49°47'N	Balsam fir	Jun. 8 – Aug. 20	1	73	3	0.0411
		Jupiter rd #3	63°27'W; 49°42'N	Trembling aspen	Jun. 8 – Aug. 20	1	73	9	0.1233
		Jupiter rd #4	63°27'W; 49°41'N	Black spruce	Jun. 8 – Aug. 20	1	73	2	0.0274
		Jupiter rd #5	63°27'W; 49°40'N	Black spruce	Jun. 8 – Aug. 20	1	73	4	0.0548
		Riv. Jupiter	63°21'W; 49°31'N	Balsam fir	Jun. 8 – Aug. 20	1	73	177	2.4247
		Pointe SO	63°24'W; 49°27'N	White spruce	Jun. 8 – Aug. 20	1	73	51	0.6986
		Riv. McDonald	63°05'W; 49°45'N	Balsam fir	Jun. 8 – Aug. 20	1	73	11	0.1507
1993	Seasonality	Lac Métis	67°48'W; 48°18'N	Balsam fir	May 31 – Aug. 23	4	340	2	0.0059
		St-Jacques-de-Leeds	71°23'W; 46°16'N	Balsam fir	May 14 – Oct. 1	4	560	0	0
1994	Diversity	Aylmer	75°52'W; 45°26'N	Balsam fir	May 31 – Aug. 18	1	80	0	0
		Lac Dumont	76°34'W; 46°03'N	Balsam fir	Jun. 2 – Aug. 17	1	77	0	0
		Mt-Laurier	75°37'W; 46°47'N	Balsam fir	Jun. 1 – Aug. 16	1	77	0	0
		Mt-Tremblant	74°11'W; 46°28'N	Balsam fir	Jun. 7 – Aug. 9	1	64	0	0
		Latuque	72°46'W; 47°19'N	Balsam fir	Jun. 7 – Aug. 8	1	63	0	0
		Lac à l’Épaule	71°11'W; 47°16'N	Balsam fir	Jun. 15 – Aug. 15	1	62	0	0
		Chute-aux-Galets	71°09'W; 48°41'N	Balsam fir	Jun. 14 – Aug. 15	1	63	0	0
		Forestville	69°06'W; 48°55'N	Balsam fir	Jun. 17 – Aug. 17	1	62	0	0
		St-Jacques-de-Leeds	71°23'W; 46°16'N	Balsam fir	Jun. 1 – Aug. 23	1	84	0	0
		Armagh	70°35'W; 46°45'N	Balsam fir	Jun. 1 – Aug. 16	1	77	1	0.013
		Pohenegamook	69°17'W; 47°37'N	Balsam fir	Jun. 15 – Aug. 16	1	63	1	0.0159
		Lac Métis	67°48'W; 48°18'N	Balsam fir	Jun. 14 – Aug. 22	1	69	0	0
		Dunière	66°47'W; 48°25'N	Balsam fir	Jun. 17 – Aug. 17	1	62	0	0
		Pellegrin	64°54'W; 48°32'N	Balsam fir	Jun. 15 – Aug. 18	1	65	2	0.0308
		Chics-Chocs	66°05'W; 48°48'N	Balsam fir	Jun. 16 – Aug. 17	1	63	0	0
		Lac Princeton	64°11'W; 49°53'N	Balsam fir	Jun. 13 – Aug. 16	1	65	0	0
1998	Trap tests	Riv Jupiter	63°21'W; 49°31'N	Balsam fir	Jun. 29 – Aug. 8	6	246	4	0.0325
2000	Exotics	St-Bruno-de-Montarville	73°21'W; 45°32'N	Red oak	May 30 – Aug. 21	1	84	0	0
		Lachenaie	73°33'W; 45°42'N	Sugar maple	May 30 – Aug. 1	1	64	0	0
		Montréal (Saraguay)	73°44'W; 45°31'N	Sugar maple	May 30 – Aug. 21	1	84	0	0
2000^a^		Cookshire	71°38'W; 45°25'N	Red/Scots pine	March 21 – May 30	8	70	0	0
2001^a^		Huntingville	71°49'W; 45°19'N	Red pine	Apr. 8 – Jun. 11	2	128	1	0.0078
				Scots pine	Apr. 8 – Jun. 11	1	64	0	0
												Bishopton	71°35'W; 45°35'N	Red pine	Apr. 8 – Jun. 11	1	64	0	0
																								Scots pine	Apr. 8 – Jun. 11	1	64	0	0
		Cookshire	71°38'W; 45°25'N	Red pine	Apr. 8 – Jun. 11	2	128	0	0																				
														Scots pine	Apr. 8 – Jun. 11	2	128	0	0										
																						Johnville	71°45'W; 45°19'N	Red pine	Apr. 8 – Jun. 11	1	64	0	0
		North Hatley	71°58'W; 45°17'N	Scots pine	Apr. 8 – Jun. 11	1	64	0	0																				
												Waterville	71°54'W; 45°16'N	Red pine	Apr. 8 – Jun. 11	1	64	0	0										
																				2002^b^		Parc Mauricie	72°58'W; 46°48'N	White pine	Jun. 21 – Aug. 2	3	129	0	0
				White spruce	Jun. 21 – Aug. 2	3	129	0	0										
		Dolbeau-Mistassini	72°14'W; 48°53'N	Jack pine	July 3 – Aug. 15	3	132	0	0
2005	Mingan Isl	Île Niapiskau	63°44’W; 50°12’N	Balsam fir	Jun. 8 – Aug. 25	1	79	0	0
		île du Havre	63°38’W; 50°13’N	Balsam fir	Jun. 8 – Aug. 24	1	78	0	0
		Grande Île	63°51’W; 50°12’N	Balsam fir	Jun. 8 – Aug. 25	1	79	0	0
2007	Anticosti Isl	Lac McCrae	64°04'W; 49°52'N	Balsam fir	Jun. 10 – July 23	2	86	4	0.0465
		Riv. Jupiter	63°20'W; 49°31'N	Balsam fir	Jun. 10 – July 23	4	172	68	0.3953
2015	*T. lineatum*	PNJC	71°19'W; 47°29'N	Balsam fir	Apr. – Aug.	4	672	0	0
^a^ Exotic surveillance for *Tomicus piniperda* in pine plantations.									
^b^ Ips attractant + 95% ethanol or α-pinene.									

**Table 2.** Data registered on the labels associated with *N. upiformis* specimens found in 45 insect collections in Quebec

**Census div/county**	**Toponym**	**Long; Lat**	**Nb. Spec.**	**Date of collection**	**Collector**	**Determinator**	**Collectiona**
Joliette	Sainte-Béatrix	73°37'00"; 46°12'00"	1	23-Jul-50	Caron, A.	S. Laplante	ORUM (CACA)
Rimouski	Rimouski	68°32'00"; 48°27'00"	1	NA^b^	NA	S. Laplante	ORUM
Terrebonne	Saint-Hippolyte-de-Kilkenny	74°01'33"; 45°55'55"	1	07-Jul-64	Venne, L.	S. Laplante	ORUM
Saguenay	Forestville	69°05'00"; 48°44'00"	1	11-Jul-50	Gills, J. R.	S. Laplante	CNC
Portneuf	Saint-Raymond	71°50'00"; 46°54'00"	1	05-Aug-33	Aubé, J.-C.	S. Laplante	LEMM (CJCA)
Île-de-Montréal	Montréal, île de	73°39'00"; 45°31'00"	1	1925	NA	S. Laplante	(CPBO)
Île-de-Montréal	Royal, mont	73°35'58"; 45°30'11"	1	02-Jul-51	Bouchard?	S. Laplante	(CPBO)
Portneuf	Saint-Raymond	71°50'00"; 46°54'00"	1	05-Aug-33	Aubé, J.-C.	S. Laplante	CCCH
Portneuf	Saint-Raymond	71°50'00"; 46°54'00"	1	10-Jun-33	Laliberté, J.-L.	S. Laplante	CINM (CJLL)
Québec	Tewkesbury	71°26'00"; 47°10'00"	4	16-Jul-51	Laliberté, J.-L.	S. Laplante	CINM (CJLL)
Québec	Québec	71°13'00"; 46°49'00"	1	25 July 19??	Laplante, J.-P. ?	S. Laplante	(CJPL)
^a^CCCA: Collection d’Armand Caron (now in ORUM); CCCH: Collection privée de Claude Chantal (Varennes); CINM: Collection de l’Insectarium de Montréal; CJCA: Collection de Jean-Charles Aubé (now in LEMM); CJLL: Collection de Joseph-Louis Laliberté (now in CINM); CJPL: Collection de Jean-Paul Laplante (now in ULQ); CNC: Canadian National Collection of Insects, Arachnids and Nematodes (Agriculture and Agri-Food Canada, Ottawa, ON); CPBO: Collection privée de Paul Bouchard; LEMM: Lyman Entomological Museum (McGill University, Sainte-Annte-de-Bellevue); ORUM : Collection Ouellet-Robert (Département de sciences biologiques, Université de Montréal, Montréal).							
^b^NA, not available.							

## References

[B4294664] Bolte A., Rahmann T., Kuhr M., Pogoda P., Murach D., Gadow K. V. (2004). Relationships between tree dimension and coarse root biomass in mixed stands of European beech (*Fagus
sylvatica* L.) and Norway spruce (*Picea
abie*s [L.] Karst.). Plant and Soil.

[B4294676] Bousquet Y., Laplante S., Hammond H. E. J., Langor D. W. (2017). Cerambycidae (Coleoptera) of Canada and Alaska: identification guide with nomenclatural, taxonomic, distributional, host-plant, and ecological data.

[B4294685] Chemsak J. A. (1996). Illustrated revision of the Cerambycidae of North America – volume I. Parandrinae, Spondylinae, Aseminae, Prioninae.

[B4295150] Desponts M., Desrochers A., Bélanger L., Huot J. (2002). Structure de sapinières aménagées et anciennes du massif des Laurentides (Québec) et diversité des plantes invasculaires. Canadian Journal of Forest Research.

[B4295189] Francoeur A. (2000). Système d'information et de gestion des échantillonnages sur la biodiversité (SIGEB) - Document technique No 8, version 3.0.

[B4295198] Gardiner L. M. (1970). Immature stages and habits of *Spondylis
upiformis* Mannerheim (Coleoptera: Cerambycidae). Pan-Pacific Entomologist.

[B4295208] Goheen D. J., Cobb Jr. F. W., Wood D. L., Rowney D. L. (1985). Visitation frequencies of some insect species on *Ceratocystis
wageneri* infected and apparently healthy ponderosa pines. The Canadian Entomologist.

[B4295218] Gosling D. C.L. (1973). An annotated list of the Cerambycidae of Michigan (Coleoptera), Part I: Introduction and the subfamilies Parandrinae, Prioninae, Spondylinae, Aseminae and Cerambycinae. Great Lakes Entomologist.

[B4295228] Jobin L. J., Desaulniers R. (1981). Résultats des pulvérisations aériennes contre l’arpenteuse de la pruche, *Lambdina
fiscellaria
fiscellaria* (Guen.), à l’île d’Anticosti en 1972 et 1973. Rapport d’information LAU-X-49F. Environnement Canada, Service canadien des forêts, Centre de recherches forestières des Laurentides, Sainte-Foy, Canada.

[B4295238] Laplante S., Bousquet Y., Bélanger P., Chantal C. (1991). Liste des espèces de Coléoptères du Québec. Fabreries.

[B4295248] Lindgren B. S. (1983). A multiple funnel trap for scolytid beetles (Coleoptera). The Canadian Entomologist.

[B4296625] MacLean D. A., Ebert P. (1999). The impact of hemlock looper (*Lambdina
fiscellaria
fiscellaria* (Guen.)) on balsam fir and spruce in New Brunswick, Canada. Forest Ecology and Management.

[B4295258] Majka C. G., Ogden J. (2010). New records of Cerambycidae in Nova Scotia. Journal of Acadian Entomological Society.

[B4295268] McCorquodale D. B., Brown J. M., Marshall S. A. (2007). A decline in the number of long-horned wood boring beetle (Coleoptera: Cerambycidae) species in Ontario during the 20^th^ century?. Journal of Entomological Society of Ontario.

[B4295287] McNamara J., Bousquet Y. (1991). Family Cerambycidae - longhorned beetles. Checklist of beetles of Canada and Alaska.

[B4295301] Potvin F., Beaupré P., Laprise G. (2003). The eradication of balsam fir stands by white-tailed deer on Anticosti Island, Québec: a 150-year process. Écoscience.

[B4295311] Sama G. (2005). Description of *Neospondylis* gen. nov. from North America and Mexico (Spondylidinae). Les Cahiers Magellanes.

[B4295321] Shibata E., Sato S., Sakuratani Y., Sugimoto T., Kimura F., Ito F. (1996). Cerambycid beetles (Coleoptera) lured to chemicals in forests of Nara Prefecture, central Japan. Annals of the Entomological Society of America.

[B4295333] Sippola A. L., Siitonen J., Kallio R. (1998). Amount and quality of coarse woody debris in natural and managed coniferous forests near the timberline in Finnish Lapland. Scandinavian Journal of Forest Research.

[B4295343] Smith G. A., Hurley J. E. (2005). First records in Atlantic Canada of *Spondylis
upiformis* Mannerheim and *Xylotrechus
sagittatus
sagittatus* (Germar) (Coleoptera: Cerambycidae). The Coleopterists Bulletin.

[B4295353] Sweeney J., de Groot P., MacDonald L., Smith S., Cocquemot C., Kenis M., Gutowski J. M. (2004). Host volatile attractants and traps for detection of *Tetropium
fuscum* (F.), *Tetropium
castaneum* L., and other longhorned beetles (Coleoptera: Cerambycidae). Environmental Entomology.

[B4295366] Webster R. P., Sweeney J. D., DeMerchant I., Silk P. J., Mayo P. (2012). New Coleoptera records from New Brunswick, Canada: Cerambycidae. ZooKeys.

[B4295387] Yanega D. (1996). Field guide to northeastern longhorned beetles (Coleoptera: Cerambycidae).

