# Supplementary figures and images for: Anticosti Island: a hot spot for Neospondylis upiformis (Coleoptera: Cerambycidae) in eastern Canada?
Source: Biodivers Data J. 2018 Jul 19;(6):e25553. doi: 10.3897/BDJ.6.e25553 (PMC6062566; doi:10.3897/BDJ.6.e25553)

## Slide 1
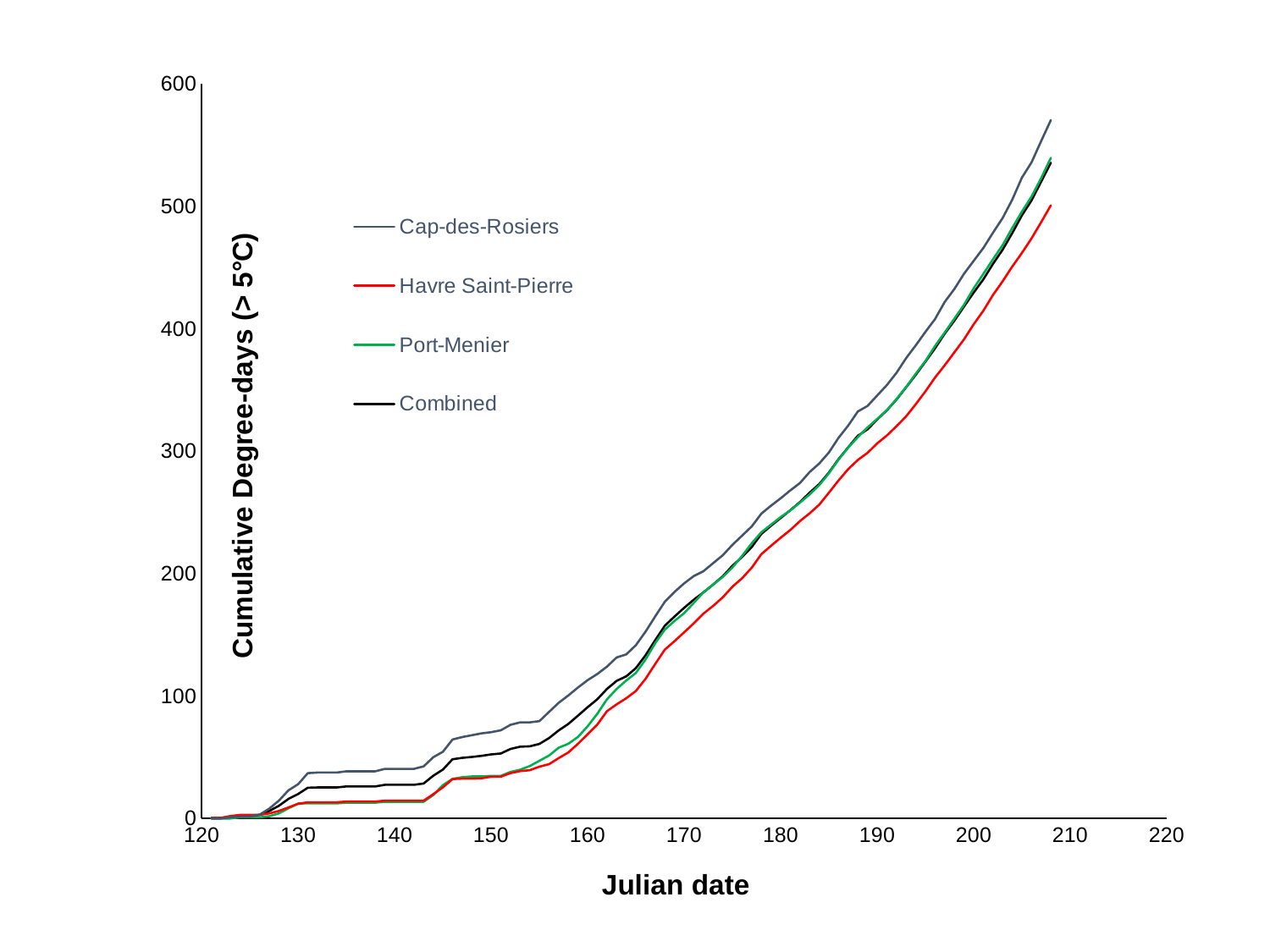

### Chart
| Category | Cap-des-Rosiers | Havre Saint-Pierre | Port-Menier | Combined |
|---|---|---|---|---|

Supplement: Supplementary material 1 — Cumulative degree-days above 5°C at Port-Menier on Anticosti Island in 2007 and comparison with data from Havre Saint-Pierre and Cap-des-Rosiers, respectively on the north and south shores of the St. Lawrence River or an average of these locations [file bdj-06-e25553-s001.pptx]
